# Orthopedic services and training at a crossroads in developing countries

**DOI:** 10.4103/0019-5413.33677

**Published:** 2007

**Authors:** Anil K Jain

**Affiliations:** Editor, Indian Journal of Orthopaedics, Professor of Orthopaedics, University College of Medical Sciences, Delhi - 110 095, India. E-mail: dranilkjain@gmail.com

Orthopedics as a specialty was started in sixth decade of last century in this country. Gradually it got established and now we are in a phase where sub-specialization is coming up. It is the right time to analyze whether we are heading in the right direction or not. It seems we are at a crossroads with lots of dilemmas.

India has always been a country of contrasts where the rich and the poor, the palace and Jhuggi (hovel) have somehow managed to exist side by side since ages. On one hand we have the poorest of the poor while at the other extreme the richest of the rich. India tops the world in possessing the highest number of blind people, and people afflicted by leprosy, tuberculosis and locomotor disabilities. On the other hand India possesses the largest number of technologists and skilled scientists.^1^

There is a vast difference between the orthopedic care in developed and developing countries. In developed countries well-organized health services have led to early diagnosis and prompt treatment of fractures, dislocations and orthopedic ailments. Road traffic and household accidents have dropped considerably due to well-established safety norms. The first aid and evacuation of the patients after an accident is prompt. The population is small, consequently less number of patients and a healthy doctor patient ratio have made the best treatment accessible to everyone possible.

On the contrary, in developing countries, the safety norms are not universally enforced, hence road traffic and household accidents are multifold. The majority of the population has to travel 50-100 km to reach the nearby health center for immediate medical help, which may be equipped only to provide elementary orthopedic care. They usually do not have enough financial resources to reach the district hospital. As a result they are treated by non-specialists or osteopaths. We see these cases in various stages, from fresh to neglected untreated fractures. A large number of them report with infected nonunion as open reduction and internal fixation has been performed in suboptimal operation theater with non-standardized implants. The non-traumatic pathologies report in various stages of natural history. The infection in general and osteoarticular TB in particular has never gone into remission contrary to developed countries.

We have a wide contrast of available health facilities. At one end the corporate hospitals in metropolitan cities are well equipped, performing state-of-the-art surgeries while at the other end no orthopedic care facilities are available at the village, taluka and tehsil level. District hospitals have orthopedic surgeons but no infrastructure and are just providing orthopedic specialty service for namesake. Medical Colleges which should have provided state-of-the-art services in specified geographic areas are examples of governmental apathy and lack of priority. Specialized trauma centers, spinal center to cater to post-traumatic and paralyzed patients are scanty. Immediately after independence a large number of medical colleges were started to produce doctors and specialists but we did not produce the required number of nurses, paramedics and technicians for effective delivery of health services.

We are spending most of the teaching time on certain aspects which are seldom practiced. The books are mainly edited by western authors and are written according to their needs. Campbell's operative orthopedics, which is most widely read, does not cover osteoarticular and spinal tuberculosis, one of the most commonly diagnosed and treated conditions in the developing countries holistically. Post-polio residual paralysis, neglected and complicated fractures and dislocations are not covered in detail. We need to develop a balance in the curriculum between our needs and recent advances. In support I would like to give one example. A laborer working in the fields reported with secondary osteoarthrosis hip to get total hip replacement done. He was advised total hip replacement (THR) in half a dozen consultations. He had pain in the right hip region but could walk for half a kilometer and he could squat albeit with pain and was able to perform the profession of a gardener. On being told that he would have to change his profession after THR as he would not be allowed to squat, he flatly refused THR saying “now I have pain walking beyond half a kilometer, however if I can't squat after the operation, my whole family will die of starvation as this is the only job I have in the rural setting”. Advance stage of AVN of femoral head in a young patient may be a case for THR by western standards, while we may still consider head preservation surgery if uniaxial motion is present. We have to develop a curriculum to make it comprehensive, need-based and which covers common psychomotor skills and concepts of evidence-based medicine. Post-qualification training should be mandatory and uniformly introduced. The evaluation method should be structured and more objective.

The objective of postgraduate orthopedic training is to provide basic biological concepts and understanding of the pathology of musculoskeletal disorders and intervention and to train him to perform basic psychomotor skills. It is also to create a curious mind for lifetime continuing education. The line between an academician and a private practitioner is getting thinner. As a result an unreasonable emphasis is on developing large surgical practices; therefore the trainee concludes that every orthopedic condition must be treated surgically. Today when many residents complete their training, they do not know how to reduce a fracture closed, apply good well-fitting plaster of Paris cast, improve fracture alignment by wedging of the casts or suspend an injured extremity in traction. Many believe that knowledge concerning the biology of fracture healing under different physiological environments has become irrelevant.^2^ Orthopedic education should not be structured to suit the marketing needs of the industry. We need more courses to discuss the principles of treatment, when to operate and when not to operate, how to choose an option out of an available list of operations rather than training for particular orthopedic operation.

We spend lots of time in evaluating trainees and trainee-related issues and little on teachers as a result the feedback to improve teaching practices is lacking. It is a biological fact that every system in the body responds to feedback. The main reason for the unprofessional and unethical deviations of some of orthopedists during and after their training is the poor example set by their teachers. Many flaws can be found in the teachers than in the students. The residents or trainees knowingly or unknowingly imitate the example that is set by many of their teachers and in due course such behavior becomes their own.^3^ Orthopedics is an artistic application of science. A surgeon starts his career where his teacher, role model and mentor leave. That is how medicine grows. Good clinicians, teachers, role models, authors, editors are groomed. Unfortunately, the quality of role models has dwindled drastically.

We live in a country with a great paradox. At one end we have tremendous intellectual capacity but we lack state-of-the-art infrastructure. The patients' rush is too heavy, the trainee is considered a workforce, as a result the education is the worst sufferer. This is true with most Government-owned medical colleges. On the other hand, we have best equipped centers in private sector with few resource persons to give conceptual teaching. These places have few patients capable of paying for surgery with hardly any opportunity for hands-on training. There is no uniformity in teaching content, and evaluation methods and examinees are induced to give different answer for similar clinical situation depending on the examiner he is facing.

The technical advances have led to a phenomenal development in orthopedics. Joint replacement, arthroscopy, spinal instrumentation have taken tremendous strides. The industry sees India as a great potential market in view of the number of patients. Even if 10% of our population (120 million) can afford high cost of treatment, it would be bigger than the whole of UK. The industry due to market-driven forces spends lots of money in scientific meetings with their sponsored speakers who will not reflect the true picture of a gadget in terms of advantages and complications. The weekend courses do not give comprehensive information about the technology. A picture is created in the minds of registrants, which suggests that particular implant is a panacea. The industry-driven projection of such a few surgeons undermines 80% orthopedic surgeons delivering yeoman service to 90% of the population in a poorly equipped district hospital. The closed treatment of a fracture is seen with low esteem. I would like to give an example to prove my point. Once while organizing a conference I received a telephone call from an industry executive to include particular speaker in the program as the said surgeon was very experienced in performing one procedure. On enquiring with the surgeon I came to know that he had performed four such operations in the last one year. It meant that a procedure would be described by a supposed expert who had only four cases as experience with less than one year of follow-up.

When a new gadget comes in the market a hype is created and the gadgets are used to treat a wide variety of clinical situations. The cases are operated with relative indications due to excitement of new technology. Later on the excitement weans off and the complications start appearing. In due course of time the rationale indications are established. This happened when rigid internal fixation of the fractures was introduced. Out of enthusiasm lots of undisplaced fractures and fractures that could have been treated closed were fixed in suboptimal O.T. conditions with inadequate training of surgeons. Hence lots of infected nonunion, implant failures were produced. A generation of orthopedic surgeons was created who were labeled “Callus haters” or “Plaster of Paris cast haters”. However, it was realized later by one and all that stable fixation with micro-motion is more biological than rigid fixation and callus is a natural method of healing. Should not we be using technology conceptually with rationale indication of surgery instead of subjecting the populations for surgery due to excitement of new technology.

## WHAT NEEDS TO BE DONE

It requires a will on the part of medical teachers, faculty of universities, medical education regulation bodies, Orthopedic Association and the government. We need to have a uniform need-based curriculum. Each medical teaching institution, whether government or private should have minimum standards of imparting education. These regulations do exist but they need to be implemented in spirit. Medical education is equally an important component of health services as patient care. Hence the teachers must be given due recognition for their contributions. There should be a system of evaluating a teacher, mentor. We have to take extra efforts in developing ourselves as a role model.

As an association we should be keeping a watch on the standard of orthopedic education and practice and liaison with the regulating bodies. The Association should become a link between past practices and future projections. We should continue to preserve the integrity of our profession and the professionalism of its members. We as an association should keep a watch on technical advances and adopt them only if it is in the best interest of our patients.^4^

We have lots of positives in this country. The sheer number of patients puts us in a commanding position to guide the orthopedic practices in the developing and the developed countries. This is possible by introducing mandatory research findings to the institutions and encouragement, incentives to the researchers. We have suffering humanity but at the same time we can create this misfortune into a boon by working hard in developing treatment modalities best suited to our population and patients at large. We lack seriousness in our policy and planning. It is time we come out of mediocrity and prepare ourselves to take on the role of a leader in orthopedics in the developing countries and the world at large.
